# Effects of Dao De Xin Xi Exercise on Balance and Quality of Life in Thai Elderly Women

**DOI:** 10.5539/gjhs.v4n1p237

**Published:** 2012-01-01

**Authors:** Patrawut Intarakamhang, Pantipa Chintanaprawasee

**Affiliations:** Department of Physical Medicine and Rehabilitation Phramongkutklao College of medicine and Hospital Bangkok 10400, Thailand Tel: 0-2354-7711 Ext 93639 E-mail:patrawutin@gmail.com; Department of Physical Medicine and Rehabilitation Naresuan University, Phitsanulok 65000, Thailand E-mail:pantipa.pmr@gmail.com

**Keywords:** Dao De Xin Xi exercise, Balance, Quality of life, Elderly

## Abstract

The objective of this study was to evaluate the effects of a 12-week Dao De Xin Xi exercise, modified short forms of Tai Chi, on balance and quality of life in Thai elderly population. Quasi-Experimental research, pretest-posttest one group design was done at Physical Medicine and Rehabilitation Department, Phramongkutklao Hospital. Thai healthy elderly women over the age of 60, requiring regular Dao De Xin Xi exercise were recruited from either patients or workers in the hospital. A 60-minute Dao De Xin Xi exercise class was set as 3 times per week for 12 weeks. At baseline and 12 weeks, participants were tested in their static balance (Single-Leg Stance Timed Test with eyes open and close), dynamic balance (Expanded Timed Up and Go (ETUG) Test). Quality of life was measured by the abbreviated Thai version of the World Health Organization Quality of Life (WHOQOL-BREF) questionnaire. Fourteen females were studied with mean age of 62.8±4.3 years old. The Single-Leg Stance Timed Test with eyes open and close, Expanded Timed Up and Go (ETUG) Test improved significantly (before versus after exercises p <0.001). Significant improvement in quality of life were also found in each 4 domains, including physical health, psychological, social relationship, and environment (before versus after exercises p =0.001, 0.001, 0.004 and 0.005 respectively). No significant improvement was found only in the right Single-Leg Stance Timed Test with eyes close (p =0.091). A three times per week for 12-week Dao De Xin Xi exercise may help Thai elderly women improve both static, dynamic balance and quality of life.

## 1. Introduction

Evolutions in medical technology lead to long-lived population, which further result in an increased number of elderly person. Thus, the elderly have to face health problems contributed from physical decline. Falls are recognized as the most common and major health problem among the elderly. It is also shown that falls are the leading cause of major injuries; for example fractures, head trauma, which account for 5-15% of falls (George, 2000) with disability and death. Falls are responsible for 70% of accidental death in persons 75 years of age and older and have psychological impacts as loss of self confidence-efficacy and then fear to fall (Scheffer *et al*, 2008) These contribute to functional decline of daily activity and low quality of life in the elderly population.

A number of studies have been performed to determine causes, risk factors, and intervention to prevent falls among elderly population (Gillespie *et al* 2003; Tinetti *et al* 1995; Thaiamwong *et al* 2008; Assantachai *et al* 2003). Exercises and other forms of physical activity; such as swimming, yoga, walking, jogging, aerobic and Tai Chi Chaun, are recommended to prevent falls(Howe *et al* 2007).

Tai Chi Chaun or Tai Chi (TCC, TC), originally developed in China is a slow and graceful Chinese exercise that includes a form of mindful meditation. TCC consists of series of individual movements linked together in a continuous manner that flow smoothly from one movement to another. TCC is now worldwide used for health benefit. A number of studies have demonstrated the beneficial effects of Tai Chi Chuan on health conditions. Wayne *et al* (2004) reviewed the studies about the improvement of vestibulopathic postural control by Tai Chi. The 24 studies supported evidences that Tai Chi may have beneficial effects for balance and postural impairments in those associated with aging. Eight of ten randomized controlled trials(RCTs) showed that Tai Chi alone, or combined with other therapies, can reduce risk of falls, improve balance and dynamic stability, increase musculoskeletal strength and flexibility, improved performance of activities of daily living (ADLs), reduced fear of falling and general improvement in psychologic well-being. Audette *et al* (2006) conducted a 12-week RCT comparing the effects of a short style of Tai Chi that was 40-45 min of the 10-movement form versus brisk walking training program in twenty six community-dwelling, sedentary elderly women on many aspects of fitness, balance and quality of life in elderly women. The participants were randomly assigned to Tai Chi Chuan (TCC; n = 11) or brisk walking group (BWG; n = 8). A separate group of elderly women was recruited from the same population to act as a sedentary comparison group (SCG; n = 8). Significant improvement was found in estimated VO_2_max in the Tai Chi Chuan group. Significant gains were also seen in the non-dominant knee extensor strength and single-leg stance time that measured balance. Kuramoto (2006) made research review about benefit of Tai Chi. There were 9 randomized controlled trials, 23 non randomized controlled trials and 15 observation studies. The author concluded that Tai Chi may lead to improved balance, reduced fear of falling, increased strength, increased functional mobility, greater flexibility, and increased psychological well-being, sleep enhancement for sleep disturbed elderly individuals, and increased cardiac functioning. Tsang *et al* (2007) conducted an RCT with thirty-eight older adults with stable type 2 diabetes comparing the benefits of a 16-week “Tai Chi for Diabetes” group with a sham-exercise-control group. Static and dynamic balance index and maximal gait speed improved significantly over time, with no significant group effects. Hackney *et al* (2008) conducted a 10-13 week RCT in thirty-three people with parkinson disease comparing a Tai Chi intervention with a non-exercise control group. The Tai Chi group participated in 20 one-hour long training sessions whereas, the control group had two testing sessions between 10 and 13 weeks apart without interposed training. The Tai Chi group improved more than the control group on the Berg Balance Scale, Unified Parkinson’s Disease Rating Scale (UPDRS), timed up and go, tandem stance test, 6-minute walk, and backward walking. Improvement in Berg Balance scores was significantly greater for the Tai Chi than the Control group. All Tai Chi participants were satisfied with the program and improvements in well-being. Almost all of the mentioned studies supported the exact evidences of balance improvement, fall prevention and also the improvement of quality of life.

Now in western, TCC has been modified in different forms (Audette *et al* 2002). A lot of studies use long form of TCC (Yang style-108 movements) which seems to be more difficult and harder to learn than short forms (8–13 movements). Short form of TCC is much easier to learn than original form and becomes better known and more favorite in urban area and city.

Dao De Xin Xi exercise was created in Bangkok, Thailand in 1998. Dao De Xin Xi exercise is a modified short form of TCC, includes 9 selected movements believed to have health benefits for 9 physiological systems in the body such as cardiovascular system, digestive system, and respiratory system etc. Dao De Xin Xi exercise is easier to perform than original TCC, softly, gently, smartly and flow slowly in a continuous repetitive left to right circular manner. Deep breathing and mental concentration are also required during exercise. In addition, each movement of Dao De Xin Xi exercise was done simultaneously with 9 easy-listening Chinese songs of Dao Xin melody arranged in Thai version, playing during exercise. The meaning and theme of all 9 songs are about mercy of mankind according to philosophical aspect, mentioned to improve mental functions by meditative effect. Today, Dao De Xin Xi exercise is well-known in almost provinces in Thailand and many participants of Dao De Xin Xi exercise got a lot of better health benefits and well being after exercise. By the way, Department of Physical Medicine and Rehabilitation, Phramongkutklao Hospital has initially adopted Dao De Xin Xi exercise for patients and interested people since the year 2006.

## 2. Objective

In Thailand, many studies of original or complete long form of Tai Chi have been done. No studies of short form of Tai Chi have been reported especially the potential effects of Dao De Xin Xi exercise. This quasi-experimental research, therefore, was the first study of Dao De Xin Xi exercise aiming to determine the effects of a 12-week exercise on balance and quality of life in Thai elderly women.

## 3. Methods

### 3.1 Population

The target population of this study was volunteers who are either patients or workers in the hospital, over the age of 60 who expressed their willingness to keep on Dao De Xin Xi exercise regularly.

### 3.2 Inclusion criteria

1) The volunteers had experience of 2 months or less Dao De Xin Xi exercise and were healthy enough for the completion of standard balance tests. 2) They could consent to participate in the study.

### 3.3 Exclusion criteria

1) The volunteers were unable to complete the standard balance tests, as specified in the study. 2) They withdrawn from the study.

### 3.4 Setting

The study setting was the activity field, Chalermprakiat building, 5^th^ floor, Phramongkutklao Hospital. A 60-minute Dao De Xin Xi exercise class was set as 3 times per week for 12 weeks, with a total of 36 times.

### 3.5 Study procedure

**Figure 1:**
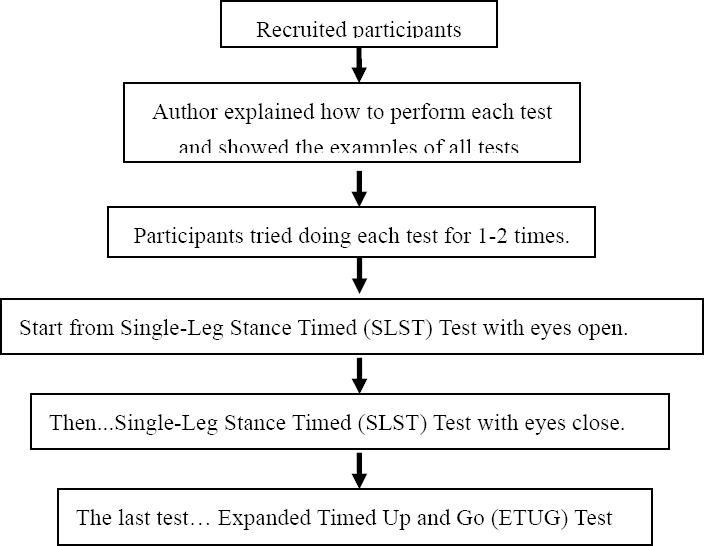
Flowchart of the balance testing procedure
  - Single-Leg Stance Timed Test (SLST) with eyes close and open
    The participant was asked to stand on a firm surface, place arms with the body, stand on one leg, and look straight ahead with eyes open. The duration of standing without trunk bending was recorded in seconds. Then, duration of single leg standing with eyes close was recorded by the same method.
  - Expanded Timed Up and Go (ETUG) Test (Figure 2.)

1) Participants were informed by the author about what Dao De Xin Xi exercise is, what to do during the study and safety of exercise. Any questions regarding the study were then answered and the written informed consent was obtained from all participants before the study.
2) The participants were asked to complete a questionnaire on demographic data including age, sex, history of illness, medications, experience of falls in the last 6 months, visual problem, and information on other forms of exercise in addition to Dao De Xin Xi.
3) The participants were tested in their balance before and after 12-week Dao De Xin Xi exercise. These tests started from Single-Leg Stance Timed Test with eyes open, followed by Single-Leg Stance Timed Test with eyes close and Expanded Timed Up and Go (ETUG) Test respectively. The author explained how to perform each tests to each participants, showed the example of tests and then let each participants try doing test 1-2 times only.
4) Conducting the test (Figure 1.)

**Figure 2:**
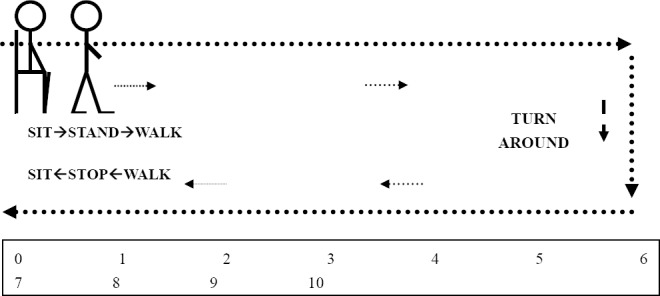
Expanded Timed Up and Go test
    The participant was asked to sit on a chair, with back against the chair and arms on the lap. When the researcher said the word “go” or gave any signal, the participant stood upright, walked at normal pace on a 10 meter- walkway to the specified mark, turned around, returned to the chair, and sat down. The stopwatch was started on the word “go” or the first signal, and stopped when the participant returned to the starting position.
5) Each participant was tested twice and the scores of both times were averaged. After the first test, each participant was allowed to rest for one minute before undertaking the next test.
6) The duration of each test was recorded in seconds.
7) Quality of life was measured by the abbreviated Thai version of the World Health Organization Quality of Life (WHOQOL-BREF) questionnaire.
8) Data were collected at baseline that was before Dao De Xin Xi exercise(experience of 2 months or less Dao De Xin Xi exercise), and at the end of the 12-week of Dao De Xin Xi exercise

### 3.6 Statistical analysis

1) Descriptive statistics including frequency, percentage, and mean were conducted to present demographic data.
2) Within participant comparison, Paired t-test was used to compare the differences in balance and quality of life before and after 12-week Dao De Xin Xi exercise. The significance level was set at 0.05.

## 4. Results

Demographic data were shown in Table 1. As shown in the table, a total of fourteen females with mean age of 64.1±4.2 years old participated in the study. Most participants had dyslipidemia and hypertension which could be improved or maintained by medications, and no walking aid was reported by all participants. 14.3% of the participants fell in the last 6 month. A half of the participants wear glasses. Prior to the study, mean duration of Dao De Xin Xi exercise was 3.9±1.4 weeks, and 35.7% of the participants performed other forms of exercise such as tennis, jogging, aerobic, and swimming.

**Table 1:** Demographic data of the participants

Demographic data	Frequency	Percentage
Sex (male/female)	0/14	0/100
Age (average/year)	64.1±4.2	
Weight (average/kg)	55.2±7.1	
Height (average/cm)	156±5.2	
Having history of illness	12/14	85.7
4 or more of concurrent medicines	2/14	14.3
Walk aid	0/14	0
Experiences of falls in the last 6 months	2/14	14.3
Visual problem(wear glasses)	7/14	50.0
Perform other forms of exercise	5/14	35.7
Mean duration of Dao De Xin Xi exercise before intervention (weeks)	3.9±1.4	

The Single-Leg Stance Timed (SLST) Test with eyes open and close and the ETUG Test have been used extensively in order to study static and dynamic balance respectively among the elderly; which reliability of the tests have also been reported.

As presented in Table 2, the SLST-Test with eyes open (right and left), the left SLST-test with eye close, and the ETUG Test improved significantly (*p* <0.001) after a 12-week of Dao De Xin Xi exercise. Although there was an increase in the right SLST-test with eye close after the exercise, no significant difference was found (*p* =0.091). During the study, no falls have been reported by the participants.

**Table 2:** Balance test before and after Dao De Xin Xi exercise (mean ± SD)

Balance tests	Before (seconds)	After (seconds)	Mean Difference (95%CI)	P-value
Right SLST-Test * (eye open)	9.72±9.17	23.94±11.65	14.22±8.59	**<0.001**
Left SLST-Test * (eye open)	8.1±6.25	20.67±6.94	12.57±5.85	**<0.001**
Right SLST-Test * (eye close)	2.89±1.10	6.11±7.22	3.23±6.62	0.091
Left SLST-Test * (eye close)	1.59±0.53	3.13±1.43	1.55±1.10	**<0.001**
ETUG Test**	17.36±1.14	15.98±1.07	-1.38±1.10	**<0.001**

* SLST-Test = Single-Leg Stance Timed Test

** ETUG Test = Expanded Timed Up and Go Test

The data about quality of life obtained from WHOQOL-BREF questionnaire(Table 3) showed that the overall quality of life improved significantly after a 12-week of Dao De Xin Xi exercise (*p* <0.001). Regarding each domain, significant improvement in quality of life were also found in each 4 domains, including physical health, psychological, social relationship, and environment (*p* =0.001, 0.001, 0.004 and 0.005 respectively).

**Table 3:** Quality of life obtained from WHOQOL-BREF questionnaire before and after Dao De Xin Xi exercise (mean ± SD)

Quality of life score	Before	After	Mean Difference (95%CI)	P-value
1. Physical	27.21±3.96	28.93±3.15	1.71±1.49	**0.001**
2. Psychological	22.00±2.91	23.64±2.27	1.64±1.69	**0.003**
3. Social relationship	11.29±2.13	12.50±1.51	1.21±1.37	**0.006**
4. Environment	31.00±4.91	33.29±3.77	2.29±2.52	**0.005**
Overall	99.07±12.55	106.71±10.21	7.64±6.06	**<0.001**

## 5. Discussion

Dao De Xin Xi exercise seems to be most likely a kind of Taoist Tai Chi that is also an exercise form of Tai Chi Chuan. Taoist Tai Chi, developed by Taoist monk in Toronto, Canada has become more popular and favorite in Western since 1970 (http://www.taoist.org/). The main foundations of Dao De Xin Xi exercise resemble those of Taoist Tai Chi such as a basic forearm rotation, a rotation of arms in front of body, a variant of the “Wave Hands like Clouds” move, repetitions of various other movements etc. Dao De Xin Xi exercise consisted of 9 movements selected from 108 movements of the Taoist Tai Chi set.

The risk factors responsible for falls could be both extrinsic, such as environmental hazards, and intrinsic, such as age-related physiologic changes especially nervous system and muscle strength (Rossat *et al*, 2003). A single fall may have multiple causes. Therefore, it is essential to evaluate and determine risk factors in order to prevent further falls. Balance problem is recognized as a major risk factor of falls and it is suggested that balance can be improved by exercise intervention (Liu and Frank, 2010; Logghe *et al*, 2010; Guan and Koceja, 2011). In addition to prevent falls, exercise can promote health status both physical and psychological functions.

### 5.1 Balance control

Balance was defined as the ability to align body segment against gravity to maintain or move the body(center of mass)within the available base of support without falling; the ability to move the body in equilibrium with gravity via interaction of the sensory and motor system(Kisner and Colby 2002).

Balancing requires concurrent processing of inputs from multiple senses, including equilibrioception (from the vestibular system), vision, and perception of pressure and proprioception (from the somatosensory system), while the motor system simultaneously controls muscle actions. The senses must detect changes of body position with respect to the base, regardless of whether the body moves or the base moves (Shumway-Cook *et al* 1988).

The study results revealed that both static and dynamic balance improved significantly after a 12-week of Dao De Xin Xi exercises. This is consistent with the studies determining the effect of TCC among elderly population. Zhang *etal* (2006) found that the elderly who performed 8-week TCC showed significant improvements in balance, flexibility, and a reduced fear of falling, when compared with the control group. Uhlig *et al* (2010) investigated the benefits of Tai Chi for fifteen patients with rheumatoid arthritis. The results revealed Tai Chi led to improved lower-limb muscle function, confidence in moving, balance and less pain during exercise and in daily life. Chyu *et al* (2010) reported sixty-one postmenopausal women with osteopenia. Subjects in Tai Chi group significantly both increased in stride width and improved in general health, vitality and bodily pain compared with those in the control group. Leung *et al*. (2011) reported systematic and meta-analytical review. The reviewed 13 RCTs showed the effectiveness of Tai Chi in improving balance in older adults and Tai chi could also reduce falls in the nonfrail elderly in the absence of other interventions. Bula *etal* (2011) conducted systematic reviewed and found the exercise including Tai Chi appears as the most promising monofactorial intervention. These are also consistent with other studies such as Schaller *et al* (1996) and Li *et al* (2008). In Thailand, Kumkate *et al* (2007) investigating the effects of original long form TCC exercise on static and dynamic balance among Thai elderly people demonstrated that the TCC group had significantly better balance control than the control group in both Single-Leg Stance Timed Test with eye open and the Expanded Timed Up and Go (ETUG) Test.

Inconsistent with some aspects of the previous research, our study found that there was an increase in the SLST-test with eyes close after the exercise, with significant difference was found only for the left leg SLST-test. However, this result was supported by Hong *et al* (2000). This may be due to the 9 individual movements of Dao De Xin Xi exercise which improved proprioceptive function. Previous studies also supported this. For example, Li *et al* (2008) examining the benefits of the 16-week TCC intervention among the elderly found that the proprioception of the knee improved significantly after the intervention, when compared to the control group. Tsang and Hui-Chan (2008) demonstrated that there were improvements in the proprioception of the knee, flexibility, and balance with eye close among the elderly who regularly performed TCC. However, because of the limited number of participants and intervention period, future studies may verify these results.

Contrary to the results of foreign research, but consistent with a Thai study conducted by Kumkate *et al* (2007), our study found that Dao De Xin Xi exercise significantly improved dynamic balance. This may be because this study used the same test as the study of Kumkate *et al* (2007), the ETUG Test(Wall *et al*, 2000), which the stopwatch was started on the word “go”, stand upright, walk at normal pace on a 10 meter-walkway to the specified mark, turn around, return to the chair, and sit down. For the foreign research, which the Time Up and Go Test was used, the walkway distance was only 10 feet. It is possible that the longer walkway may lead to the more sensitivity in balance test which possibly results in higher performance.

It’s not yet clear which mechanism plays role in balance improvement. The combination of improvement of proprioception, increased trunk, leg muscle flexibility and strength is more likely to be than only single specific mechanism. The improvement of vestibular function and the possible linkage of psychological well-being may probable be the adjuvant mechanism of balance improvement (Wayne *et al* 2004).

### 5.2 Quality of life

The results of quality of life showed that the overall quality of life improved significantly after a 12-week of Dao De Xin Xi exercise. The significant improvements were also found in each 4 domains, including physical health, psychological, social relationship, and environment. This is consistent with Deschamps *et al* (2009) and Taylor-Piliae *et al* (2006). Some explanations are responsible for this. The first may be due to the improvement in balance control after exercise. Second, as Dao De Xin Xi is considered to be an aerobic exercise, is easy to perform with melodious Dao Xin song providing meditation and mental training, these may result in psychological relaxation. The final explanation is social relationship among the group of elderly during the exercise.

Philosophically, the meanings of 9 songs, simultaneously playing with exercise are intended to help the people regain original nature of goodness in human such as taking care of each other etc. This itself may improve psychological being and social relationship included as parts of quality of life.

### 5.3 Limitation of the study

The first is the small sample size which may be a limitation in terms of statistical significant. Second, the generalization of the findings is limited by characteristics of the participants: female, healthy, have no history of illness, and moderate to high quality of life.

Recommendations for further study are as follows. First, studies in a larger sample size of elderly population and also in elderly population with health problems; such as coronary heart disease, diabetes, hypertension, and at risk of falls, should be conducted in order to further create useful exercise guideline. Second, there should be a study examining the association between the improvement in balance and quality of life using more reliable instrument, i.e. computerized dynamic posturography. Finally, there is a need for additional studies evaluating the potential effects of Dao De Xin Xi exercise on other physical and mental conditions; such as bone density, cognitive function, and sleep quality in elderly.

## 6. Conclusion

A 60-minute Dao De Xin Xi exercise 3 times per week for 12 weeks may help Thai elderly female participants improve both static, dynamic balance, and quality of life.
